# Diagnostic Challenges in Cecal Bascule: A Case Report

**DOI:** 10.7759/cureus.112992

**Published:** 2026-07-20

**Authors:** Sherif Elgohary, Emad Addin AbuOsba, Baha H Abuajameia, Ahmed H Elgohary, Hesham Aljohary

**Affiliations:** 1 College of Medicine, Qatar University/Hamad Medical Corporation, Doha, QAT; 2 College of Medicine, Qatar University, Doha, QAT; 3 College of Medicine, QU Health, Qatar University, Doha, QAT; 4 College of Medicine, Tbilisi State Medical University, Tbilisi, GEO; 5 Acute Care Surgery, Hamad Medical Corporation, Doha, QAT

**Keywords:** cecal bascule, cecal volvulus, computed tomography, exploratory laparotomy, large bowel obstruction

## Abstract

Cecal bascule is a rare subtype of cecal volvulus characterized by anterior folding of the cecum without axial torsion. Its nonspecific presentation often makes early diagnosis challenging, and delayed recognition may lead to bowel ischemia, perforation, or closed-loop obstruction. We report the case of a 77-year-old female patient with type two diabetes mellitus who presented with five days of right-sided abdominal pain, progressive abdominal distension, constipation, vomiting, and anorexia. Laboratory investigations showed leukocytosis, markedly elevated C-reactive protein, mild lactic acidosis, hypokalemia, and hyponatremia. Contrast-enhanced computed tomography (CT) demonstrated a distended anteriorly displaced cecum with findings suggestive of incomplete cecal volvulus, favoring cecal bascule. Emergency surgical exploration was performed, initially planned as diagnostic laparoscopy but converted to midline laparotomy due to marked colonic distension. Intraoperatively, the cecum, ascending colon, and transverse colon were severely dilated, with the cecum folded anteriorly and a constricting band causing obstruction. The obstruction was relieved by mobilization of the cecum and ascending colon, with no bowel resection required. The patient recovered postoperatively and was discharged in stable condition. This case highlights the importance of considering cecal bascule in patients presenting with acute large bowel obstruction and emphasizes the role of early CT diagnosis and timely surgical intervention.

## Introduction

Intestinal obstruction remains a common and clinically significant surgical emergency, most frequently caused by postoperative adhesions, incarcerated hernias, malignancies, and inflammatory strictures [1]. Less commonly, large bowel obstruction may result from volvulus, which accounts for a minority of cases but carries a higher risk of ischemia and perforation when diagnosis is delayed. Within this subset, cecal bascule represents an exceptionally rare and underrecognized etiology, accounting for only a small fraction of cecal volvulus cases and an even smaller proportion of all intestinal obstructions [2].

Cecal volvulus is a rare clinical entity with an average incidence of 2.8-7.1 per million people per year, accounting for one to two percent of all large bowel obstructions. Cecal bascule is the rarest type of cecal volvulus, accounting for 5-20% of all cases [3]. It occurs when the cecum folds anterosuperiorly over a horizontal axis in a manner resembling a see-saw, from which the term “bascule” is derived in French. Unlike classical cecal volvulus, which involves axial twisting of the bowel and mesentery, cecal bascule is characterized by folding of the inferior pole of the cecum without mesenteric torsion. This abnormal folding creates a potential point of luminal obstruction that may impede the passage of colonic contents. In the presence of adhesions between the cecum and ascending colon, particularly with a competent ileocecal valve, the condition can progress to a closed-loop obstruction. Ongoing accumulation of gas from colonic bacterial activity subsequently leads to progressive cecal distension, venous congestion, compromised arterial perfusion, and ultimately ischemia, gangrene, or perforation if left untreated [3-5].

Patients with cecal bascule typically present with features of acute or subacute large bowel obstruction, including progressive abdominal distension, colicky abdominal pain, nausea, vomiting, and failure to pass flatus or stool. However, the clinical presentation is often nonspecific and indistinguishable from other causes of intestinal obstruction, which makes early recognition difficult and frequently leads to delayed diagnosis [3-5]. Diagnosis is primarily radiological, with contrast-enhanced computed tomography (CT) being the modality of choice, demonstrating anterior and superior displacement of a distended cecum with a characteristic folding configuration and absence of the classic mesenteric “whirl sign” seen in axial volvulus [6,7]. Management is largely surgical, as nonoperative approaches are associated with a 95% failure rate and risk of progression to ischemia or perforation [8].

We report a case of cecal bascule presenting as acute intestinal obstruction treated at Hamad Medical Corporation, Qatar.

## Case presentation

In 2016, a 77-year-old woman with a past medical history of type two diabetes mellitus presented to the emergency department complaining of right-sided abdominal pain for five days. The pain was associated with distension, anorexia, constipation, and five episodes of vomiting. The pain was severe, dull, non-radiating, and unrelated to food intake. She also noted lower urinary tract symptoms for one day. As for past surgical history, it was only significant for a cesarean section.

On physical examination, the patient appeared to be in pain, indicated by her lying on her left side. She was tachycardic, tachypneic, and afebrile. Examination of the abdomen showed generalized distention with right upper quadrant, flank, and iliac fossa tenderness in addition to guarding. Bowel sounds were absent. A digital rectal examination was unremarkable. Laboratory investigations demonstrated leukocytosis (white blood cell count: 15.1 × 10³/µL), elevated C-reactive protein (332 mg/L), mild lactic acidosis (2.2 mmol/L), hypokalemia (2.8 mmol/L), and mild hyponatremia (133 mmol/L) (Table 1).

**Table 1:** Baseline laboratory investigations upon admission. Values are presented with standard SI units. Reference ranges may vary slightly depending on the specific institutional laboratory and analysis equipment utilized. WBC: White Blood Cell; CRP: C-Reactive Protein; K+: Potassium; Na+: Sodium.

Laboratory Parameter	Patient Value	Reference Range	Interpretation
White blood cell (WBC) count	15.1 × 10³/µL	4.0 – 11.0 × 10³/µL	Elevated (leukocytosis)
C-reactive protein (CRP)	332 mg/L	< 5.0 mg/L	Severely elevated
Potassium (K+)	2.8 mmol/L	3.5 – 5.0 mmol/L	Low (hypokalemia)
Sodium (Na+)	133 mmol/L	135 – 145 mmol/L	Mildly low (hyponatremia)
Lactate (lactic acid)	2.2 mmol/L	0.5 – 2.0 mmol/L	Mildly elevated (mild lactic acidosis)

**Figure 1:**
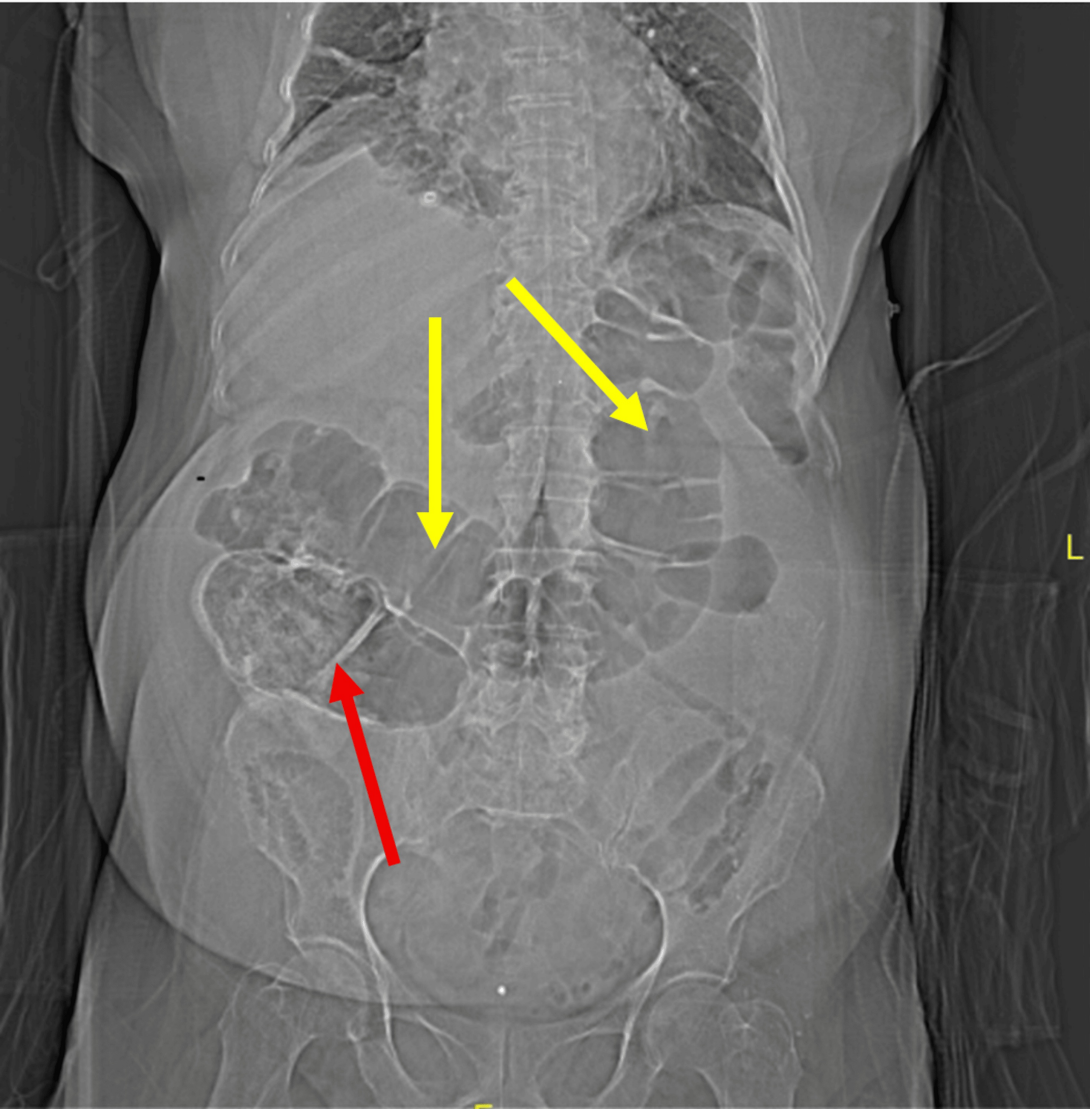
Contrast-enhanced CT abdomen showing anterior displacement and distension of the cecum. Preoperative abdominal radiograph demonstrating a low-mechanical large bowel obstruction with a massively distended, gas-filled cecal loop displaced into the central abdomen (red arrow). The yellow arrow highlights the pronounced distension of the ascending colon, confirming significant proximal large bowel dilation.

**Figure 2:**
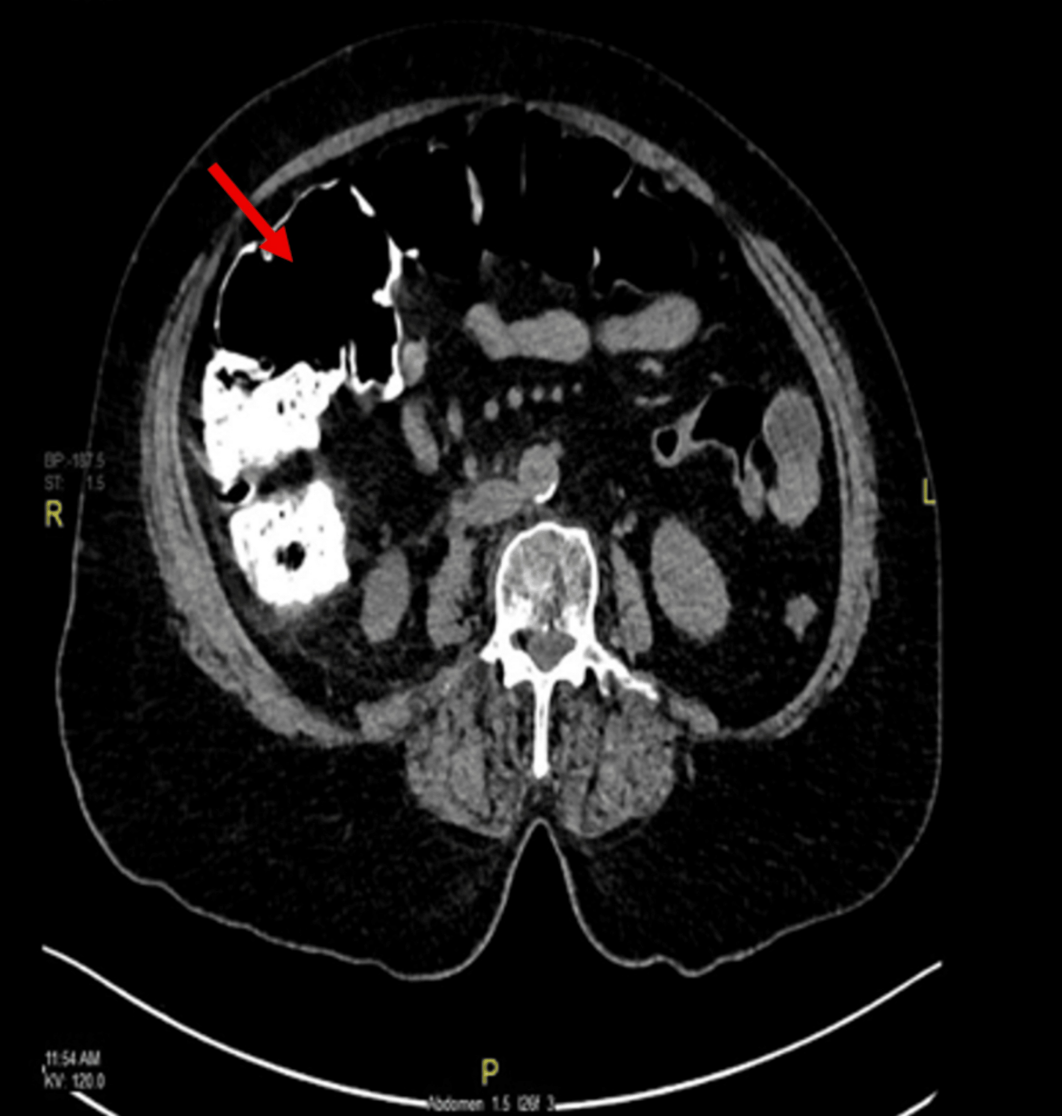
Contrast-enhanced CT abdomen demonstrating features consistent with cecal bascule. Axial CT image of the abdomen revealing a massively distended cecum (indicated by the red arrow) displaced from its normal anatomical position in the right lower quadrant and folding upward, highly indicative of a cecal bascule.

A contrast-enhanced CT scan of the abdomen was ordered, which revealed a distended cecum located anteriorly in the central part of the abdomen. The ascending colon showed a thickened, edematous wall; however, it remained in a normal position. Gas was noted extending up to the splenic flexure, while distally, the colon was collapsed. These findings were suggestive of incomplete cecal volvulus in favor of cecal bascule (Figures 1, 2).

The patient's worsening symptoms and imaging findings warranted the decision to proceed with emergency surgical exploration. The patient was informed regarding the procedure and the possibility of bowel resection, and informed consent was obtained.

**Figure 3:**
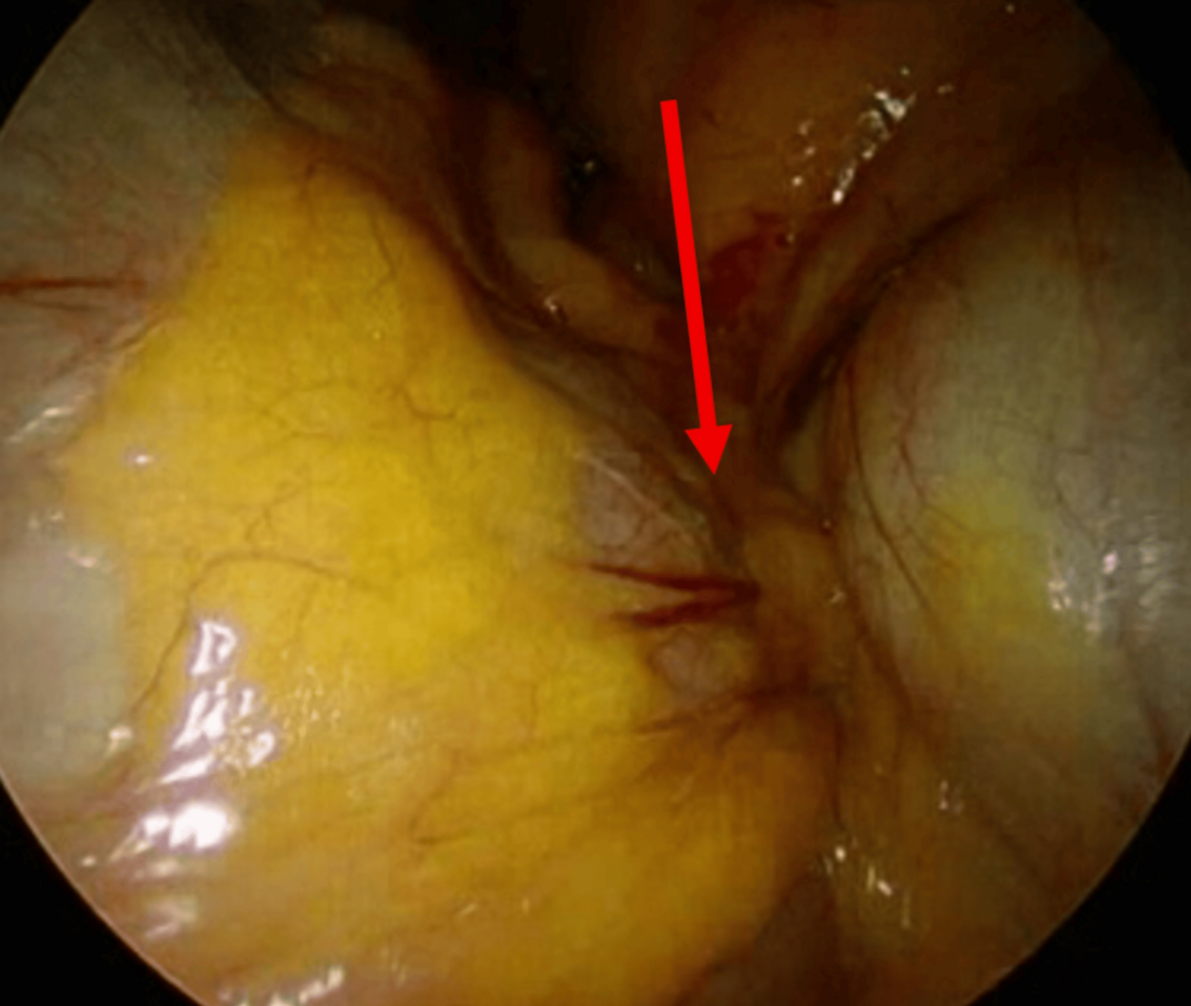
Laparoscopic identification of an obstructing cecal bascule. The red arrow points directly to the sharp constriction band formed at the compression interface where the mobile cecum has folded anteriorly and medially over the fixed ascending colon. This mechanical folding creates a characteristic flap-valve mechanism resulting in bowel obstruction.

The initial surgical plan was diagnostic laparoscopy; however, due to marked colonic distention and difficulty proceeding safely, it was converted to a midline exploratory laparotomy. Intraoperatively, there was no free intraperitoneal fluid or evidence of bowel ischemia that would warrant resection. The cecum, ascending colon, and transverse colon were severely dilated. The cecum was folded on its own axis, anteriorly, towards the center of the abdomen with a constricting band surrounding the organ. The cecum and ascending colon were mobilized, relieving the obstruction. No bowel resection was performed, hemostasis was secured, and the abdomen was closed (Figure 3).

**Figure 4:**
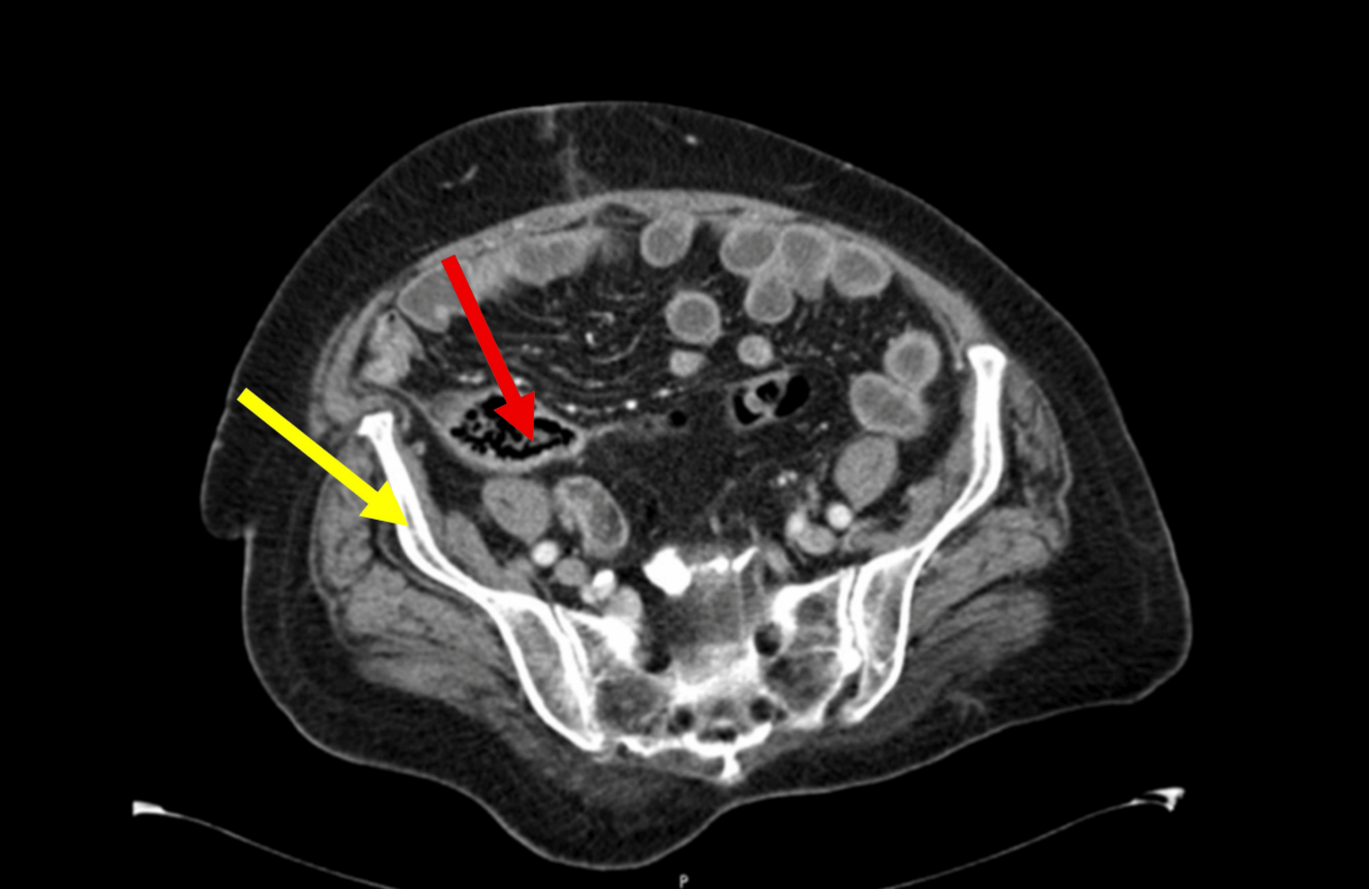
Postoperative CT abdomen showing the cecum low in position at the pelvic inlet and appears less distended. Axial pelvic CT slice obtained on follow-up demonstrating the cecum (red arrow) located deep within the pelvis. The structure is positioned immediately adjacent to the right pelvic bone (yellow arrow). No evidence of large bowel obstruction or colonic dilation is present in this scan.

Postoperatively, the patient was admitted intubated to the surgical intensive care unit for ventilatory and hemodynamic support. She received intravenous fluids, electrolyte correction, glycemic control, gastric ulcer prophylaxis, and broad-spectrum antibiotics with piperacillin-tazobactam. The patient was successfully extubated the following day and gradually improved with conservative postoperative management. Her abdominal distension subsided, and bowel function returned progressively. She was subsequently discharged in stable condition with outpatient follow-up. A postoperative CT scan showed the cecum in the pelvis with adequate resolution of the bascule (Figure 4). Over a 10-year follow-up period, the patient remained well with no documented recurrence of cecal bascule, supporting durable resolution following surgical reduction.

## Discussion

Cecal bascule is an exceptionally rare subtype of cecal volvulus. Although the cecum is normally an immobile secondary retroperitoneal structure, congenital or acquired malfixations or adhesions may increase its mobility and predispose it to volvulus. While axial torsion (type I) and loop volvulus (type II) comprise at least 80% of cecal volvulus cases, cecal bascule (type III) represents the least common variant. Cecal bascule is characterized by superomedial folding of the cecum and ileocecal valve, resulting in displacement towards the right upper quadrant. It is slightly more common in males and typically affects individuals between 40 and 70 years of age [3]. Due to its rarity, the risk factors for cecal bascule are not fully understood. Reported predisposing factors include previous abdominal surgery, chronic constipation, high-fiber intake, ileus, and distal colonic obstruction [8].

The clinical presentation of cecal bascule is often nonspecific, contributing to a diagnostic challenge. Common manifestations include abdominal pain, abdominal distension, nausea, vomiting, and altered bowel habits. In postoperative patients, symptoms may be misattributed to more common conditions such as postoperative ileus, thereby obscuring the diagnosis. In advanced cases, patients may develop hemodynamic instability, confusion, and features of intestinal ischemia [9]. As there is no axial rotation of the colon, mesenteric vascular compromise is uncommon, and patients typically present with a less severe clinical course.

Radiological evaluation plays a pivotal role in diagnosis. Although plain abdominal radiographs may demonstrate a markedly dilated cecum with an “inverted teardrop” appearance, these findings are nonspecific. Therefore, CT is the imaging modality of choice. In contrast to type I and II cecal volvulus, the cecal bascule lacks the classic “whirl sign” on CT scans due to the absence of mesenteric twisting [3]. Characteristic CT features include an anteriorly displaced, gas-distended cecum folded upon itself and positioned anterior to the ascending colon.

Furthermore, CT is valuable for detecting complications such as pneumatosis intestinalis, pneumoperitoneum, mesenteric stranding, and bowel ischemia. Although it is the initial diagnostic tool of choice, with a reported sensitivity of 61%, some cases are diagnosed only during exploratory laparotomy [3]. Early recognition through cross-sectional imaging, however, remains essential to facilitate timely intervention and reduce morbidity [8].

Surgical management remains the mainstay of treatment for cecal bascule, given the high failure rate of conservative approaches and the risk of progression to ischemia, perforation, or recurrence [10]. However, due to its rarity, there is no standardized or universally accepted treatment algorithm. Most reported cases are managed operatively, with right hemicolectomy or ileocecal resection and primary ileocolic anastomosis considered the most definitive approach, particularly in the presence of ischemia or non-viable bowel, as resection effectively eliminates the risk of recurrence. Alternatively, when the bowel remains viable, cecopexy serves as an option for surgical reduction and fixation. However, this approach carries significant drawbacks, including high recurrence and mortality rates, with no evidence demonstrating clinical superiority over other surgical techniques [5,10].

To the best of our knowledge, this is the first reported case of cecal bascule in the Gulf region. This case highlights the diagnostic challenges associated with this rare entity, particularly its nonspecific presentation and potential mimicry of more common postoperative conditions. It underscores the importance of maintaining a high level of suspicion and vigilance for timely diagnosis. Increased clinician awareness may reduce the risk of potentially life-threatening complications.

## Conclusions

Cecal bascule is a rare and often underrecognized cause of large bowel obstruction with nonspecific clinical features that may delay diagnosis. This case highlights the importance of maintaining a high level of clinical suspicion in patients presenting with abdominal distension, pain, constipation, and vomiting, particularly when computed tomography findings suggest anterior displacement and folding of the cecum.

Early radiological diagnosis and timely surgical intervention are essential to prevent devastating clinical complications such as ischemia, perforation, and closed-loop obstruction. Prompt operative management in this patient resulted in an excellent clinical recovery and successful structural resolution without the need for bowel resection.
